# Technical efficiency of public health centers in three districts in Ethiopia: two-stage data envelopment analysis

**DOI:** 10.1186/s13104-018-3580-6

**Published:** 2018-07-13

**Authors:** Firew Tekle Bobo, Mirkuzie Woldie, Muluemebet Abera Wordofa, Gebeyehu Tsega, Tesfamichael Alaro Agago, Kifle Wolde-Michael, Nuraddis Ibrahim, Elias Ali Yesuf

**Affiliations:** 1 Department of Public Health, Wollega University, Nekemte, Ethiopia; 2 Department of Health Policy and Management, Jimma University, Jimma, Ethiopia; 3 Fenot Project of Harvard T.H. Chan School of Public Health, Boston, USA; 4 Department of Population and Family Health, Jimma University, Jimma, Ethiopia; 5 Department of Epidemiology, Jimma University, Jimma, Ethiopia; 6 Department of Biomedical Science, Jimma University, Jimma, Ethiopia; 7 CIHLMU Center for International Health, Ludwig-Maximilians-Universität München, Munich, Germany

**Keywords:** Technical efficiency, Scale efficiency, Health centers, Inputs, Output, Data envelopment analysis

## Abstract

**Objective:**

The aim of the study was to measure technical and scale efficiency of public health centers in three districts of Jimma zone, Ethiopia. A two-stage data envelopment analysis was used. First, we estimated technical and scale efficiency of the health centers. In the second stage, institutional and environmental factors were against technical efficiency of the health centers to identify factors associated to efficiency of the health centers.

**Results:**

Eight out of the 16 health centers in the study were found to be technically efficient, with an average score of 90% (standard deviation = 17%). This indicates that on average they could have reduce their utilization of all inputs by about 10% without reducing output. On the other hand, 8 out of 16 health centers were found to be scale efficient, with an average scale efficiency score of 94% (standard deviation = 9%). The inefficient health centers had an average scale score of 89%; implying there is potential for increasing total outputs by about 11% using the existing capacity/size. Catchment population and number of clinical staff were found to be directly associated with efficiency, while the number of nonclinical staff was found to be inversely associated with efficiency.

## Introduction

A number of countries in Africa have been inefficient in the use of available resources. From the total fund allocated to the health system worldwide, 20–40% of all health resources being wasted. The achievement of national and international health development targets requires not only an increase in funding, but also efficient use of available resources and greater equity in financing and accessing quality health care [1–4]. In Ethiopia, the government is majorly concerned with addressing access and coverage issues and there is lack of empirical evidence on the level of efficiency of health centers in the overall delivery of health services [4–6].

Health centers play a central role in Primary Health Care Unit, which are a vital part of Ethiopia’s public health system. Significant investments are directed at improving the quality and equitable delivery of health services provided at health center level, with a strategic emphasis on crucial and interrelated elements—accessibility, affordability, and sustainability [7].

However, not much has been done to assess the efficiency of those health facilities. Health center is deemed efficient, if it can produce the maximum possible output using a given amount of input. Data envelopment analysis (DEA) is an important tool that is used to measure efficiency of decision-making units (Health centers in this case). DEA is a methodology directed to frontiers rather than central tendencies.

Therefore, using data envelopment analysis (DEA) framework, this study aimed to determine technical efficiency, scale efficiency of individual health centers in three districts of Ethiopia. Moreover, we also estimated the amount of input reduction and/or output increases needed to make inefficient health centers efficient. Finally, we aimed to identify factors associated with efficiency of health centers.

## Main text

### Methods

#### Study design and setting

Institution based cross sectional study design was employed. All health centers in three districts; namely, *Mana*, *Kersa* and *Seka chekorsa* of Jimma zone were included in the study.

#### DEA conceptual framework

To assess differences in the productive efficiency of health centers, we used DEA, a mathematical programming based method that converts multiple input and output measures into a single summary measure of productive efficiency. DEA is based on relative efficiency concepts proposed by Farrell but Charnes et al. extended and developed Farrell’s approach [8, 9].

Overall this model, measures the ability of the health center to produce a given level of output using the minimum amount of input or alternatively the maximum amount of output using a given amount of input. The formula is given by [8, 9].

$$ Max\, h_{o} \, = \, \frac{{\mathop \sum \nolimits_{r = 1}^{s} u_{r} y_{ijo}  }}{{\mathop \sum \nolimits_{i = 1}^{m}   v_{i} x_{ijo}  }} $$

Subject to:

$$ \frac{{\mathop \sum \nolimits_{r = 1}^{s} u_{r} y_{i}  j_{o} }}{{\mathop \sum \nolimits_{i = 1}^{m}   v_{i} y_{i}  j_{o} }} \, \le \,1, \quad j\, = \,1, \ldots  j_{o} , \ldots n $$

$$ u_{r} \, \ge \,0 r\, = \,1, \ldots , s\, \,and\, \,v_{i}  \, \ge \,0, \quad i\, = \,1, \ldots m $$

y_rj_: amount of output r from health center j, x_ij:_ amount of input i to health center j, u_r_: weight given to output r, v_i_: weight given to input i, n: number of health centers, s: number of outputs, m: number of inputs.

*Constant returns to scale (CRS) model* Measured using Charnes, Cooper and Rhodes (CCR) DEA model. Measures health centers ability to produce expected/required amount of output from a given amount of input. The formula is given by;

$$ Max \,h_{o} \, = \, \mathop \sum \limits_{r = 1}^{s} u_{r} y_{rjo} .$$

Subject to:

$$ Max \,h_{o} \, = \, \mathop \sum \limits_{r = 1}^{s} u_{r} y_{rjo} \, = \,1 $$

$$ Max\, h_{o} \, = \,\mathop \sum \limits_{r = 1}^{s} u_{r} y_{r} \, - \, \mathop \sum \limits_{r = 1}^{s} v_{i} x_{ij} \, \le \,0 \quad  j\, = \,1, \ldots , n $$

$$ u_{r} ,v_{i}  \, \ge \,0 $$

*Variable returns to scale (VRS) model* Measured using Banker, Charnes and Cooper (BCC) DEA model. This model is effective to assess efficiency of the health centers when all inputs proportionally increase and we face different level of output production.

$$ Max\, h_{o} \, = \, \mathop \sum \limits_{r = 1}^{s} u_{r} y_{rjo} \, + \,z_{jo} $$

Subject to:

$$ Max \,h_{o} \, = \, \mathop \sum \limits_{r = 1}^{s} v_{ir} x_{ijo} \, + \, z_{jo} \, = \,1 $$

$$ Max \,h_{o} \, = \, \mathop \sum \limits_{r = 1}^{s} u_{r} y_{r} \, - \, \mathop \sum \limits_{r = 1}^{s} v_{i} x_{ij} \, + \,z_{jo} \, \le \,0 \quad  j\, = \,1, \ldots , n $$

$$ u_{r} ,v_{i}  \, \ge \,0 $$

#### Data collection procedure

The instrument was prepared after reviewing different literatures. The Ethiopian standard for health centers requirement [10] and other literatures [11–14] were used to prepare the document review check list in order to collect the data from the health centers. The contents of the document review checklist (data collection instrument) includes, input and output data and environmental factor such as catchment population of those health centers in the year of 2013/2014.

#### Data analysis

First, descriptive statistics of all input and output variables were calculated by using Stata 13. The mean, standard deviation (SD), minimum and maximum values of all input and output variables were presented. Subsequently, the technical efficiency, scale efficiency scores and input reduction and/or output increases were computed using the DEA Programme, version 2.1 (DEAP 2.1) developed by Coelli [8]. Health facilities that assume the “best practice frontier” are assigned an efficiency score of one (or 100%) and are said to be technically efficient compared to their peers. The efficiency of the health facilities below the efficiency frontier is measured in terms of their distance from the frontier. The inefficient health facilities are assigned a score between one and zero. The larger the score the more efficient a health facility is [8].

In the second stage, the estimated technical efficiency scores obtained from the DEA was considered the dependent variable and regressed against the set of institutional and environmental variables using a Tobit model.

### Results

#### Descriptive statistics of input and output data

The health centers used 25 Health officers, 106 clinical Nurses, 30 Midwives, 29 laboratory technicians, and 19 Druggists all together to provide care for 163,698 outpatients, 11,077 pentavalent three times for children, four and more antenatal care (ANC) for 12,279 pregnant women, delivery care for 9504 mothers, family planning for 33,249 women all together. Table 1 shows the difference between efficient (eight health centers) and inefficient eight health centers by the inputs used and outputs they produced.

**Table 1:** Inputs used and outputs produced among efficient and inefficient health centers

Inputs	Efficient health centers			Inefficient health centers		
	Mean	SD	Sum	Mean	SD	Sum
Nonclinical staff	8.9	5	71	8.6	2.4	69
Clinical staff	13.3	3.8	106	13.1	2	105
Outputs						
Outpatient visits	13,848.5	6940	110,788	6614	3862.6	52,910
Pentavalent 3 times	767.6	321.6	6141	617	313	4936
ANC four and more	975.6	271.9	7805	559	226.1	4474
Delivery	716.2	362.1	5730	471.7	150	3774
Family planning	2429.5	1325.3	19,436	1726.6	1341	13,813

#### Efficiency analysis

The overall average score for technical efficiency was 77% with SD of 16%, CRTS technical efficiency 90% (SD = 17%), for VRTS technical efficiency the average score was 94% (SD = 11%), and for scale efficiency (SE) the average score was 94% (SD = 9%). Table 2 presents scores for constant returns to scale, variable returns to scale, scale efficiency, and returns to scale of 16 health centers.

**Table 2:** Presents, constant return to scale, variable return to scale, scale efficiency and return to scale values of each health center

DMU	CRTS	VRTS	SE	RTS
HC01	0.89	0.92	0.98	Decreasing
HC02	1	1	1	Constant
HC03	0.97	1	0.97	Decreasing
HC04	0.94	0.96	0.98	Increasing
HC05	0.5	0.75	0.67	Increasing
HC06	1	1	1	Constant
HC07	1	1	1	Constant
HC08	0.91	1	0.91	Increasing
HC09	0.68	0.87	0.78	Increasing
HC10	1	1	1	Constant
HC11	1	1	1	Constant
HC12	1	1	1	Constant
HC13	1	1	1	Constant
HC14	1	1	1	Constant
HC15	0.96	0.96	0.99	Decreasing
HC16	0.54	0.63	0.85	Increasing
Mean	0.90	0.94	0.94	
STD deviation	0.17	0.11	0.09	

#### Constant return to scale (CRTS)

From the total of 16 health centers involved in the analysis eight (50%) had a constant return to scale technical efficiency of 100%, and the rest 8 health centers were constant return to scale inefficient. Out of the 8 CRTS inefficient health centers 4 (25%) had a score between 91 and 99.99%, two had a score between 50 and 59%, one a score of 68%, and another 1 health center had a score of 87% constant return to scale technical efficiency.

#### Variable return to scale (VRTS)

The findings of VRTS model shows that 10 (62.5%) of the health centers had a score of 100%, and the rest 6 (37.5%) were found to be inefficient. Among the inefficient 3 (18.75%) had a score between 91 and 99.99% and other three different health centers had a score between 60 and 90%, two scored 87 and 75%, and one scored 63%.

#### Scale efficiency

Table 2 shows 8 (50%) of health centers in the three Woredas of Jimma Zone are scale inefficient. Implying that they are either too small or too large. Increasing returns to scale was the predominant form of scale inefficiency.

#### Tobit regression analysis

Table 3 shows the Tobit regression model results, that identifies the factors that affect efficiency of public health centers.

**Table 3:** Presents Tobit regression model result

*Tobit regression*				
Number of obs = 16				
LR χ^2^(5) = 24.95				
Prob > χ^2^ = 0.0001				
Log likelihood = 12.59				

Efficiency	Coef.	P	(95% conf. interval)	
Catchment population	7.80E−06	0.013	2.03E−06	1.36E−05
Outpatient visit	7.04E−06	0.120	− 2.17E−06	1.62E−05
Clinical staff	0.06063	0.000	0.036432	0.084827
Nonclinical staff	− 0.02501	0.039	− 0.04847	− 0.00156
Age	3.64E−05	0.998	− 0.02584	0.025911
_cons	− 0.10668	0.762	− 0.86388	0.650522
/sigma	0.079686		0.044311	0.115062

*Obs. summary*				
0	Left-censored observations			
8	Uncensored observations			
8	Right-censored observations			at efficiency = 1

### Discussion

The findings of the study revel that eight (50%) of health centers were technically inefficient. Though, technical inefficiency is widely prevalent according to studies conducted in some of sub-Saharan African countries, the finding of this study is a little bit higher than others. The majority of studies in those countries present above 50% of technical inefficiency, for instance 65% of public health centers in Ghana [13], 59% of peripheral health units in Pujehun district of Sierra Leone [12], 56% of Public Health Centers in Kenya [15], 78% of Public Health Centers in Ghana [16] were all found to be technically inefficient.

The average technical efficiency of the eight inefficient health centers was 90% with a standard deviation of 17%. This indicates that on average they could have reduce their utilization of all inputs by about 10% without reducing output.

Differences of technical and scale efficiency results of this study with other findings in sub-Saharan Africa countries discussed above could be attributed to different reasons. This might be due to the differences of health care system and their performances. Moreover, health insurance scheme in Ghana, Kenya and Sierra Leone enable people to use health services—promotion, prevention, treatment and rehabilitation—without incurring financial hardship, which induces demand for health care and increases output produced by the health facilities.

Increasing the amount of outputs requires an increase in the demand for health care. Since, input needs of health center are standardized, reduction of inputs is not an option. In order for 8 inefficient facilities to become efficient as a group, they would have needed to increase their outpatient department visits by 23,177 (77%), family planning by 4390 (14.5%), immunization by 1010 (3.3%), ANC 4^+^ by 970 (3.2%) and delivery care by 694 (2.3%).

The second DEA stage analysis identified two significant factors which have positive association with efficiency. This factors were the size of catchment population and clinical staff of the health centers. On the other hand, nonclinical staff was found to affect efficiency negatively.

In conclusion, only half of the health centers were found to be scale efficient. There was barely a difference between the eight efficient health centers and the other eight inefficient health centers, in the amount of health care workers they used. However, clients/patients who were served at the efficient health centers were more than twice in number than those clients who were served at inefficient health centers. Considering the scarce resource available to the health sector, the findings indicate that performance improvement measures have to be taken.

## Limitations of the study

- The analysis reported in this article is based on health centers inputs and outputs data for 2013/2014. Much has changed since 2013/2014, particularly in terms of the country’s socioeconomic and health development. The results are not meant to uncritically inform current decision-making processes, but rather to illustrate the potential value of such efficiency analyses.
- Limitations of the study may include, DEA attributes any deviation from the “best practice frontier” to inefficiency, while some could be due to statistical noise, e.g. epidemics or measurement errors.
- Expenditures on pharmaceuticals and non-pharmaceutical supplies and other nonwage expenditures among the inputs were not included in the study due to the lack of data.

## Abbreviations

CRS: constant return to scale
VRS: variable return to scale
SE: scale efficiency
DEA: data envelopment analysis
DMU: decision making units
WHO: World Health Organization
PHC: Primary Health Care
PHCU: Primary Health Care Units
MOH: Ministry Of Health (Federal Democratic Republic of Ethiopia)
